# The Effect of Live Spontaneous Harp Music on Patients in the Intensive Care Unit

**DOI:** 10.1155/2013/428731

**Published:** 2013-11-27

**Authors:** Ann Marie Chiasson, Ann Linda Baldwin, Carrol Mclaughlin, Paula Cook, Gulshan Sethi

**Affiliations:** ^1^Arizona Center for Integrative Medicine, University of Arizona, Tucson, AZ 85724-5153, USA; ^2^Department of Physiology, University of Arizona, Tucson, AZ 85724-5051, USA; ^3^Laboratory for the Advances in Consciousness and Health, Department of Psychology, University of Arizona, Tucson, AZ 85721-0068, USA; ^4^School of Music, University of Arizona, Tucson, AZ 85721-0004, USA; ^5^Department of Cardiothoracic Surgery, University Medical Center, University of Arizona, Tucson, AZ 85724-5071, USA

## Abstract

This study was performed to investigate the effect of live, spontaneous harp music on individual patients in an intensive care unit (ICU), either pre- or postoperatively. The purpose was to determine whether this intervention would serve as a relaxation or healing modality, as evidenced by the effect on patient's pain, heart rate, respiratory rate, blood pressure, oxygen saturation, and heart rate variability. Each consenting patient was randomly assigned to receive either a live 10-minute concert of spontaneous music played by an expert harpist or a 10-minute rest period. Spontaneous harp music significantly decreased patient perception of pain by 27% but did not significantly affect heart rate, respiratory rate, oxygen saturation, blood pressure, or heart rate variability. Trends emerged, although being not statistically significant, that systolic blood pressure increased while heart rate variability decreased. These findings may invoke patient engagement, as opposed to relaxation, as the underlying mechanism of the decrease in the patients' pain and of the healing benefit that arises from the relationship between healer, healing modality, and patient.

## 1. Introduction

Use of complimentary and alternative medicine (CAM) as an adjunct to healing has been studied in postoperative cardiac patients in a few settings. Kshettry et al. [1] found that using CAM as an adjunct treatment in a pre- and postoperative setting is both feasible and useful for decreasing patient pain and tension. They studied the effects of guided imagery and gentle massage before operation, music for two days after surgery, and gentle massage after leaving intensive care, versus patient standard care. The authors found that, of these CAM therapies, music therapy was associated with reduced pain and tension in early recovery from cardiac surgery.

Music has been used as a CAM adjuvant for healing and symptom management. Research on this topic has increased over the last decade including four published reviews in the Cochrane database in the past 5 years. The data published to date suggest that listening to music affects multiple physiological parameters, especially those related to the autonomic nervous system (ANS). Bradt et al. reviewed the evidence for music therapy with cancer patients and concluded that music therapy may have beneficial effects on anxiety, pain, quality of life, heart rate (HR), respiration rate (RR), and blood pressure (BP) in cancer patients [2]. Another study by Huang et al. revealed that cancer patients who listened to sedative music reported less pain than those receiving analgesic alone [3]. Listening to music also appears to benefit patients receiving mechanical ventilation by influencing their HR, RR, and anxiety [4, 5]. In addition, Bradt et al. [2], in their review on the effects of listening to music by patients with coronary artery disease, concluded that listening to music “may have a beneficial effect on their BP and HR.” Music can also be effective in reducing anxiety in patients with myocardial infarction (MI) and there may be a slight benefit in reducing pain [6]. In another Cochrane review by Cepeda et al., it was concluded that music reduced pain, and in those patients who experienced reduced pain, the required dose of opioid analgesics was also decreased [7].

One possible mechanism by which listening to music can exert beneficial effects is through increased relaxation. White [8] studied the effects of relaxing music on the cardiac ANS after an acute MI. They compared music in a restful environment, rest alone, and normal therapy with no intervention. No difference in BP was found between the three groups, but heart rate variability (HRV) increased in the rest group and the music group, while anxiety decreased in the music group. These results support listening to music as a way of preventing the deleterious effects of the stress response.

Acute stress can activate the immune system and cause inflammation through the complement cascade [9]. On the other hand, chronic stress can impair immune function and delay wound healing [10]. It is important that when people are in a state of healing their immune systems are functioning optimally, that is, neither overreacting nor underreacting. Therefore environmental stress should be kept to a minimum. Unfortunately, in hospitals, due to the high level of personnel activity and noise from monitors, this is not always possible. For this reason, the concept of whether music increases relaxation in the hospital environment is of interest. For this study, spontaneous, live harp music was specifically chosen because live harp music has been previously investigated with positive findings in the hospital setting [11–13].

One small study (*N* = 17) addressed live harp music in a postoperative thoracic unit and found that live harp music decreased BP and HR and increased oxygen saturation (O_2_SAT) [12]. In addition, Sand-Jecklin and Emerson [14] researched the impact of live relaxing harp music intervention on patients' experience of pain, anxiety and muscle tension after admission to the hospital with an emergent medical or traumatic illness. Thirty-one patients reported significant reductions in pain, anxiety and muscle tension and significant reductions in RR and systolic BP, while no significant difference in HR or diastolic BP. So far, no effects of harp music on patients' HRV have been reported except for a study on premature infants by Kemper and Hamilton [11]. In this study harp music did not significantly alter HRV. However, only 8 infants were monitored and they were divided among 3 groups only one of which experienced harp music.

The purpose of this study was to investigate the effect of live, spontaneous harp music on individual patients in an intensive care unit (ICU), either pre- or postoperatively to determine whether it would serve as a relaxation modality, decreasing HR, BP and respiration rate and increasing HRV and whether, based on previous results, it would reduce self-reported pain. Each consenting patient was randomly assigned to receive either a live 10-minute concert of spontaneous music played by an expert harpist or a 10-minute rest period. The type and severity of the diseases were similar between the two groups.

## 2. Materials and Methods

### 2.1. Experimental Design

This study used a case control design with pre- and postmeasurements on a convenience sample of all patients admitted to an academic ICU. The University of Arizona Institution Review Board approved the study design prior to its commencement. Before starting the study, a power analysis was performed to determine a statistically appropriate group size. Using previous data comparing HRV before and during self-administration of another alternative therapy, a group size of 25 was calculated. Power was estimated assuming standard *α* values (95% confidence limits or *α* = 0.05). The calculated power was 0.87 and was above the sufficient statistical power of 0.8.

All patients in the ICU who were able to consent, or who had a family member who could consent, were invited to participate, regardless of diagnosis or pre- or postoperative status. Patients who agreed was consented and the study performed. Measurements were made on patients between the hours of 10 am and 3 pm. The intervention was a 10-minute live, spontaneous harp session in the patients' rooms. The same harpist, a professional harpist and harp teacher, was used for all patients in the experimental group. She played spontaneous improvised music with the intention of helping the patients heal. For the control group, the patients were left in their rooms for 10 minutes and advised to relax during that time period. Each participant (cases and control group) was given CD of the harpist at the end of the session as a thank you and as incentive for participation in the study.

### 2.2. Experimental Measures

Heart rate variability is a noninvasive measure of the complementary relationship between the sympathetic and parasympathetic branches of the ANS [15]. Instruments used for recording HRV analyze the signal by means of time domain or frequency domain (spectral analysis) to quantify the variability in HR that exists in a given recording. Time domain parameters include the standard deviation of the interbeat interval (IBI), SDRR, which provides a gross measure of HRV, and the root mean square of successive differences in IBI (RMSSD), which reflects the parasympathetic activity of the ANS.

Prior to the harp or control session, a plethysmograph (pulse sensor) was clipped to the subject's ear lobe for HRV measurements. When HRV was recorded, the patients were resting, either lying in bed or sitting on a reclining chair, and they were asked not to speak during the measurement to prevent movement artifacts from confounding the data. The sensor was attached to a computer via an emWave PC (HeartMath LLC) device that output inter-beat interval (HR) data to a text file that was analyzed in greater detail using a freeware HRV program, http://kubios.uef.fi/. Algorithms within the software program provide interactive interpretation of waveforms for the raw interbeat interval data collected. During analysis any abnormal beats were manually removed from the data. Artifacts due to slight movements of the patients were also eliminated. In cases in which too much data are eliminated, some HRV indices cannot be properly estimated. We therefore introduced an artifact percentage threshold of 10% to our analysis [16]. Data files from which more than 10% of the data had to be eliminated were excluded from the analysis.

**Figure 1:**
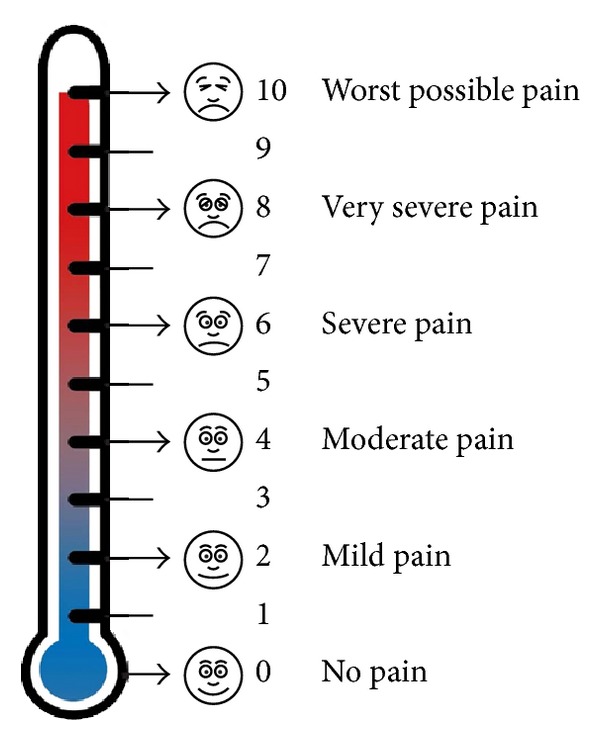
The enclosed material was prepared by Northeast Health Care Quality Foundation (NHCQF), the Medicare Quality Improvement Organization (QIO) for Maine, and New Hampshire and Vermont, under contract with the Centers for Medicare & Medicaid Services (CMS), an agency of the U.S. Department of Health and Human Services. The contents presented do not necessarily reflect CMS policy.

During the 5-minute period while HRV was being recorded, systolic and diastolic BP, O_2_ SAT, RR, and HR were collected from the monitors or from the patient, directly. Patient pain report, as per the thermometer visual pain scale, (Figure 1) was also reported. All measurements were repeated after the patients in the experimental group had heard 10 minutes of harp music, and after the patients in the control group had rested for another 10 minutes. No medications were given to patients in either group during the interval between initial and final measurements. In a given week, data were collected from either the music group or the control group in order to prevent the live harp music from affecting the control patients in adjacent rooms. The data were analyzed by a member of the research team blinded as to the identity of the data groups.

### 2.3. Statistical Analysis

For gender comparison between groups, a 2 × 2 chi square test was used for *P* value <0.05. Student *t*-tests were run to test for significant differences between the two groups for baseline values of all physiological parameters. For data sets that failed the normality test, the Mann-Whitney rank sum test was used. Paired Student *t*-tests were run for control and for music groups to determine the treatment effect, pre versus post, for all parameters. For data sets that failed the normality test, the Wilcoxon signed rank test was used. If the *P* value were less than 0.05, the statistical comparison would be considered significant.

## 3. Results and Discussion

Statistical analysis demonstrated no significant difference between control and music groups for patient age or gender. These results are presented in Table 1. In addition, there was no significant difference between baseline values of control and those of music groups for any of the measured physiological parameters. With regards to pain, there was a 27% reduction in patient-reported pain after the music (a decrease of 0.8 on a pain scale of 0–10 (*P* = 0.005)). There was no significant difference in pain for the control group pre- and postrest session. Seven of the 50 patients in the control group and 11 out of the 50 patients in the music group were asleep during the experiment and so no pain scores could be obtained from those patients. Our statistical analysis showed that there were no significant differences regarding age or gender between the two groups from whom we obtained pain scores.

The other physiological parameters (HR, O_2_ SAT, BP, RR, and HRV) did not show any significant differences pre and postmusic or rest period. These data are presented in Tables 2 and 3. Values of N for HRV are less than fifty as some data could not be analyzed due to arrhythmias and anomalies. Although there was no significant difference regarding age between the control and music group patients from whom HRV data were obtained, there was a significant gender difference (*P* = 0.002, chi square = 9.62). It is unlikely that this difference affects the HRV data because gender differences in HRV disappear after age 50 [17]. The average ages of the two groups from whom HRV data were obtained were 58.8 and 56.6.

Most of the patients were at a fairly low level of pain before the harpist played, the average being 3 on a scale from 0 to 10. The finding of a 0.8 decrease in the pain scale may seem low, yet it actually represents a 27% reduction in pain. The fact that a significant reduction in pain was experienced despite the initial low level of pain speaks to the effectiveness of the music. A significant reduction in pain in this environment is a valuable finding; the perception of pain by the patients is an important aspect of patient care and healing. Patients' pain is often not adequately addressed in clinical settings and this study has shown that a simple noninvasive intervention such as a live harp session can have a positive impact on pain and outcome. Pain medications used in ICU setting have been associated with significant side effects. These include nausea, vomiting, respiratory depression, constipation, hallucinations, and disorientation. This supports the recommendation of use of safe adjunctive therapies for pain control that do not have these side effects.

While our study reveals that spontaneous live harp music significantly decreased patient perception of pain, the music did not significantly affect RR, HR, HRV, BP, or O_2_ SAT when values were averaged over all patients in a given group. Since the study took place in the ICU, most of the patients were taking medications that would affect HR and BP (such as cardiac medications including beta-blockers) and RR and pain (such as opiates). In an effort to reduce the impact of this study as much as possible on nursing care in the ICU, patient medication data were not collected from the electronic medical records of the participants. That element would have added an entirely different aspect to our study, because it would have compelled nurses to stop their work and check their patient's chart with us, thus disrupting the flow of work in the ICU. For this reason the data analysis could not be adjusted to account for medications that impact HR, BP, and RR. Had patient data been stratified into cardiac and pain medications this may have led to different findings. Since pharmacological interventions were already in place to keep the patients' outcome measures as low and stable as possible this also means that the effects of the harp music on outcome measures were probably underestimated. Although it would be interesting to repeat this study on patients not taking medications that affect the ANS, this is not clinically or ethically feasible for patients in pain.

Notably, the HRV measures in our music group trended toward a decrease after intervention, although these findings were not statistically significant. The small size of the effect was probably due to the fact that many of the outcome variables were most likely modified or dampened from the pharmacological interventions the patients were receiving. That being said, these trends are potentially an interesting finding. A larger study would likely illuminate this result, especially if the data were stratified by pharmacologic interventions.

Another trend was that systolic BP increased slightly in the music group after intervention despite pharmacologic control. This finding contrasts with another result [14] in which a slight but significant decrease in BP was observed in patients who listened to slow tempo, relaxing harp selections on the Celtic harp. However, in our study each patient may have been affected differently on an autonomic level, with differing degrees of arousal and relaxation. A large variation in patient response is likely, because the harpist played music for each person consisting of the combination of tempos that she perceived was most appropriate for them. Previous studies [18, 19] have shown that the underlying tempo of music may have an effect on HR and BP. In those experiments an arousal effect was observed proportional to the speed of the music, with slower rhythms inducing relaxation and faster rhythms promoting sympathetic stimulation.

The idea that varying tempos of music may have different effects on the ANS highlights the concept that the decreased pain experienced by some of the patients in our study may not be from relaxation, but from engagement with the music. This idea is consistent with the observed slight reduction in HRV. This response may be evidence of the impact of a healing dynamic within the healer-patient relationship. Our harpist played directly to whatever she was sensing within the patient in order to enable healing through the music and through her presence with the patient. One aspect of this study that is unique is the participation of a master harpist and composer who is able to play spontaneous compositions directed specifically towards each patient's needs. This type of playing may have captured the effect not of relaxation by the patient but of engagement in a healing relationship. Patients reported they felt better and more energized. This may be reflected in the decreased pain and also slight rise in BP. It would be interesting to complete another study with taped harp music and see how this in would be compared to the live sessions and controls.

These findings, if sustained and amplified in a larger study, could lead to an interesting discussion about what other measures could be introduced into the ICU to engage patients, that is, an increase in volunteers, reading to patients or having other health related practitioners visit patients in the ICU. In fact, perhaps focusing on the patient's attention is a significant factor in pain control. It seems that the human aspect of this focus is important, as many of the patients were already watching television when they were asked to prepare for the intervention. The control patients continued to watch television, an engaged focus with no relationship. The harpist's intention was to fully engage the patient, not to make them relax. The intervention engaged the patients visually (for those awake), auditorially and socially. Perhaps engaging patients in a multisensorial way will reduce pain and decrease the use of pain medication in an ICU-like setting.

There were limitations to the study. Firstly, the patients were all in the ICU, some preoperatively, some postoperatively, and some were in the ICU for the only reason of needing a monitored ICU bed when there was ICU over-flow. Therefore not all the patients had a cardiac diagnosis. This is the practical reality of how the ICU unit runs in the hospital in which this study was performed. The decision was made to include all patients as potential candidates for the music or control groups to mimic reality in the event that this intervention was to be adopted by this hospital.

Another confounding variable was that other family members or acquaintances often joined the participants. In order to mimic reality as much as possible, all individuals in the music or the control group were taken as they were without asking anyone to leave the room. However, the effect of other people in the room could have altered the patient's relaxation state and healing response. Since the intervention needs to be feasible for routine usage, the presence of patient visitors remains a confounding variable that cannot be assessed. A third limitation to the study was that the 2-group design did separate out the effects of the actual music from those of the nonverbal interaction between the harp player and the patients. Inclusion of a third group of patients listening to recorded harp music would show which of these two factors is of primary importance in reducing patient pain perception.

Lastly, since participation in the experiment was voluntary, patients who did not like harp music could decline. There are data indicating that the individual taste of patients affects how effective music is as an intervention [20]. Our study showed that live harp music decreased pain by 27% in a volunteer sample of people who liked harp music. If live harp music is offered to patients as a standard therapy, music preference would not matter as only those who liked the harp would agree. However, if these results were extrapolated to live music in other settings, such as the waiting area for the operating room, the impact of the music would be influenced by personal preferences of those listening.

Despite the limitations, the impact of this study on the environment of the ICU is important. One of the clinical nurse specialists who oversaw the ICU wrote this comment to us. “As so many of us, I truly believe in the healing properties of music and would love nothing more than to share this with others. This harp study has been a wonderful experience for both our patients and staff,” (personal communication).

## 4. Conclusion

This study reveals that spontaneous live harp music significantly decreased patient perception of pain by 27% in a ICU setting, but on average RR, HR, HRV, and O_2_ SAT were not significantly affected. Possibly each patient was affected differently on an autonomic level, with differing degrees of arousal and relaxation, because the harp piece for each individual containing a different combination of tempos that the harpist perceived was most appropriate for them. The reduction in patient's perception of pain supports the introduction of live harp music into the ICU as a non-invasive means to reduce patient pain.

## Figures and Tables

**Table 1:** Music and control group comparison.

Parameter	Music gr.	Control gr.	*P* value
*N*	50	50	
Average age (years)	65.29 (13.8) ^1^	59.05 (19.3)	NS
Percent male^2^	58%	72%	NS

^1^(Standard deviation) of age.

^2^Chi square = 2.9 with 1 df, *P* = 0.1.

**Table 2:** Experimental parameters for music group pre- and postharp session.

Parameter	*N* ^1^	Preharp session (SD)	Postharp session (SD)	*P* value
Respiration rate (br/min)	48	19.8 (6.0)	19.4 (5.9)	NS
Oxygen saturation (%)	49	95.8 (4.7)	96.20 (4.3)	NS
Systolic BP (mmHg)	50	112.0 (23.3)	113.3 (22.8)	NS
Diastolic BP (mmHg)	50	62.1 (16.0)	61.2 (13.7)	NS
Heart rate (bpm)	50	84.4 (16.6)	83.0 (16.1)	NS
SDRR2^2^ (ms)	23	37.4 (28.3)	33.7 (28.5)	NS
RMSSD^3^ (ms)	23	48.3 (43.7)	42.2 (40.4)	NS
Pain (0–10)	39	3.0 (3.3)	2.2 (2.7)	0.005

^1^Values of *N* for HRV (SDRR and RMSSD) are less than 50 as some data could not be analyzed due to arrhythmias and anomalies. Values of *N* for pain are reduced due to inability for some patients to self-report pain.

^2^SDRR: standard deviation of the RR interval in the HRV monitoring.

^3^RMSSD: the root means square of successive differences of the RR interval.

**Table 3:** Experimental parameters for control group pre- and postrest period.

Parameter	*N* ^1^	Prerest period (SD)	Postrest period (SD)	*P* value
Respiration rate (br/min)	48	19.3 (4.8)	18.8 (4.6)	NS
Oxygen saturation (%)	49	96.2 (3.2)	96.3 (3.0)	NS
Systolic BP (mmHg)	48	116.7 (21.0)	115.7 (20.5)	NS
Diastolic BP (mmHg)	48	62.6 (11.4)	61.2 (12.0)	NS
Heart rate (bpm)	48	76.7 (9.6)	76.7 (9.8)	NS
SDRR^2^ (ms)	27	34.3 (29.2)	32.7 (29.1)	NS
RMSSD^3^ (ms)	27	45.5 (44.5)	43.2 (42.9)	NS
Pain (0–10)	43	2.5 (3.0)	2.5 (3.0)	NS

^1^Values of *N* for HRV (SDRR and RMSSD) are less than 50 as some data could not be analyzed due to arrhythmias and anomalies. Values of *N* for pain are reduced due to inability for some patients to self-report pain.

^2^SDRR: standard deviation of the RR interval in the HRV monitoring.

^3^RMSSD: the root means square of successive differences of the RR interval.
